# Ablation of *Htra1* leads to sub-RPE deposits and photoreceptor abnormalities

**DOI:** 10.1172/jci.insight.178827

**Published:** 2025-02-10

**Authors:** Pooja Biswas, DaNae R. Woodard, T.J. Hollingsworth, Naheed W. Khan, Danielle R. Lazaro, Anne Marie Berry, Manisha Dagar, Yang Pan, Donita Garland, Peter X. Shaw, Chio Oka, Takeshi Iwata, Monica M. Jablonski, Radha Ayyagari

**Affiliations:** 1 Shiley Eye Institute, UCSD, La Jolla, California, USA.; 2 The Hamilton Eye Institute, University of Tennessee Health Science Center, Memphis, Tennessee, USA.; 3 Ophthalmology and Visual Sciences, University of Michigan, Ann Arbor, Michigan, USA.; 4 The National Institute of Sensory Organs (NISO), NHO Tokyo Medical Center, Tokyo, Japan.; 5 Harnly LLC, Bethesda, Maryland, USA.; 6 Functional Genomics and Medicine, Division of Biological Science, Nara Institute of Science and Technology, Ikoma, Nara, Japan.

**Keywords:** Genetics, Ophthalmology, Mouse models, Neurodegeneration, Retinopathy

## Abstract

The high-temperature requirement A1 (HTRA1), a serine protease, has been demonstrated to play a pivotal role in the extracellular matrix (ECM) and has been reported to be associated with the pathogenesis of age-related macular degeneration (AMD). To delineate its role in the retina, the phenotype of homozygous *Htra1*-KO (*Htra1^–/–^*) mice was characterized to examine the effect of *Htra1* loss on the retina and retinal pigment epithelium (RPE) with age. The ablation of *Htra1* led to a significant reduction in rod and cone photoreceptor function, primary cone abnormalities followed by rods, and atrophy in the RPE compared with WT mice. Ultrastructural analysis of *Htra1^–/–^* mice revealed RPE and Bruch’s membrane (BM) abnormalities, including the presence of sub-RPE deposits at 5 months (m) that progressed with age accompanied by increased severity of pathology. *Htra1^–/–^* mice also displayed alterations in key markers for inflammation, autophagy, and lipid metabolism in the retina. These results highlight the crucial role of HTRA1 in the retina and RPE. Furthermore, this study allows for the *Htra1^–/–^* mouse model to be utilized for deciphering mechanisms that lead to sub-RPE deposit phenotypes including AMD.

## Abstract 2

In this manuscript, we characterize and describe the ocular consequences of genetically removing the high-temperature requirement A1 (*Htra1*) gene in the mouse retina.

## Introduction

Several ECM components are implicated in vision loss in age-related macular diseases such as the most common one, age-related macular degeneration (AMD) (1, 2). AMD is currently the leading cause of irreversible blindness in the aging population and is characterized by the degeneration of the RPE–Bruch’s membrane (RPE-BM) and photoreceptors (3–6). The 2 forms of AMD, exudative/wet and atrophic/dry, display prominent clinical features and cause substantial vision loss (3). Exudative/wet AMD features include leakage of blood or fluid (macular neovascularization) (7) into the subretinal space (3). In contrast, features of nonexudative/dry AMD include the accumulation of sub-RPE deposits (i.e., drusen, basal linear deposits) (3, 8) and geographic atrophy (9, 10). To help uncover the genetic associations of AMD, genome-wide association studies have identified many genetic loci associated with risk for AMD (11). Among these, the *ARMS2/HTRA1* on chromosome 10q26 is a major risk locus for AMD development (11). However, it is currently challenging to distinguish the role of *ARMS2* and *HTRA1* as they are in linkage disequilibrium (12–14).

The high-temperature requirement A1 (*HTRA1*) serine peptidase 1 gene encodes for a 51 kDa serine protease that is expressed in many tissues, including the retina (15–18). In the retina, HTRA1 is secreted from the RPE into the BM, which comprises the ECM (19, 20). One of the most well-known physiological roles of HTRA1 is its ability to cleave numerous ECM proteins, including EFEMP1 and C1QTNF5/CTRP5 that are involved in monogenic retinal degenerative diseases such as Malattia Leventinese/Doyne honeycomb retinal dystrophy (ML/DHRD) (20) and late-onset retinal degeneration (L-ORD) (21), respectively. Of great interest is that certain phenotypic features of these aforementioned retinal diseases, such as the presence of sub-RPE deposits and/or RPE atrophy, are similar to clinical observations in patients with AMD (22). Because of its role in the retinal ECM, recent studies have suggested a potential role for HTRA1 in the development of BM abnormalities and RPE pathology (12, 13, 15, 21, 23–25).

While the involvement of *HTRA1* in AMD has been reported, the mechanisms underlying *HTRA1* contribution to pathology remain unknown. Several studies using model systems have suggested that increased *HTRA1* expression contributes to retinal pathology in patients (23, 26–29). However, William et al. recently reported that the diminished expression of *HTRA1* in the RPE of AMD donor eyes can also cause pathology (24). Thus, this prompted us to assess the effect of ablation of *Htra1* on retinal structure and function in *Htra1^–/–^* mice. Our results show that *Htra1^–/–^* mice display reduced photoreceptor function with rod degeneration and RPE atrophy accompanied by sub-RPE deposits. Thus, the ablation of *Htra1* affects overall retina and RPE integrity, and it recapitulates distinct pathological features observed in macular degeneration.

## Results

### Htra1^–/–^ mice display impaired photoreceptor function.

**Figure 1:**
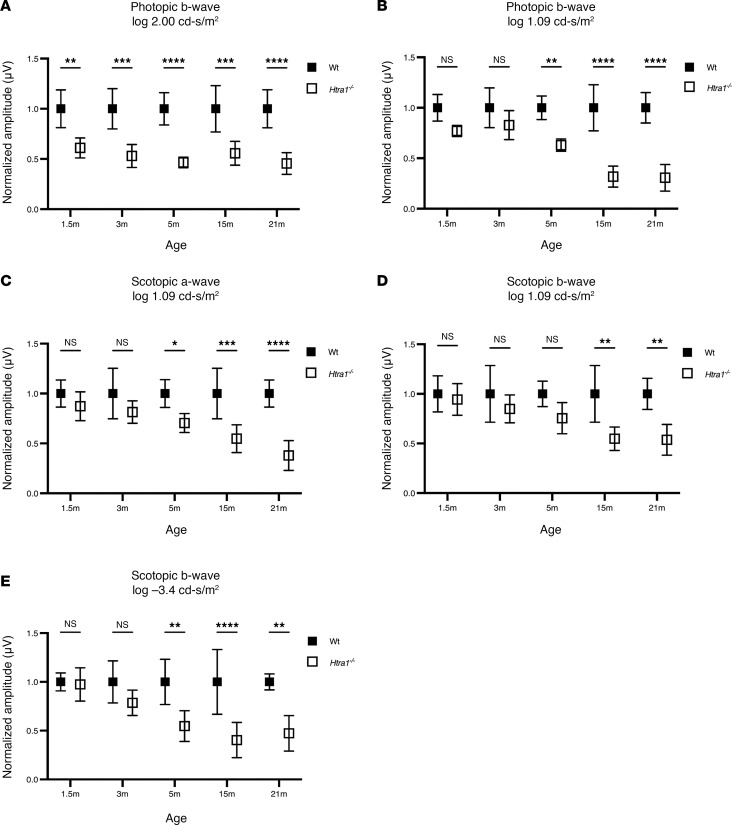
Photoreceptor function in *Htra1^–/–^* mice. (**A**–**E**) Mean photopic and scotopic responses of 1.5, 3, 5, 15, and 21m *Htra1^–/–^* mice compared and normalized to age-matched WT mice at 1.09 log cd·s/m^2^, 2.00 log cd·s/m^2^, and –3.5 log cd·s/m^2^ stimulation intensities. *n* = 5 mice per age point and genotype. Two-way ANOVA followed by the Tukey-Kramer for pairwise comparisons, with *P* values adjusted using the Bonferroni Correction. Age-matched comparisons are displayed. *P < 0.05, **P < 0.01, ***P < 0.001, ****P < 0.0001.

After genotyping and confirming the expression of *Htra1* mRNA in WT and *Htra1^–/–^* mice (Supplemental Figure 1, A–D; supplemental material available online with this article; https://doi.org/10.1172/jci.insight.178827DS1), retinal function and integrity was assessed in *Htra1^–/–^* mice compared with WT. To assess the effect of loss of *Htra1* on retinal function with age, full-field electroretinography (ffERG) was performed on 1.5-month (1.5m), 3m, 5m, 15m, and 21m *Htra1^–/–^* mice compared with all age matched (1.5m, 3m, 5m, 15m, and 21m old WT) control mice (Figure 1, A–E, and Supplemental Figure 2). The photopic response of *Htra1^–/–^* mice was significantly reduced at 1.5m (*P* = 0.0188, log 1.09 cd·s/m^2^) (Figure 1B). Subsequently, photopic responses in *Htra1^–/–^* mice steadily decreased with age from 3m to 21m compared with age-matched WT controls (Figure 1, A and B). To assess rod function, scotopic a- and b-wave responses were recorded at various stimulation intensities (Figure 1, B–E). Interestingly, *Htra1^–/–^* rod mediated responses at log –3.5 cd·s/m^2^ (*P* = 0.0010) and log 1.09 cd·s/m^2^ (*P* = 0.0056) show significant loss from 5m compared with WT (Figure 1, C and E). These results suggest that loss of *Htra1* affects the function of both rod and cone photoreceptors with a measurable loss in photopic response around 1.5m while a decrease in scotopic response was evident at 5m.

### Altered cone morphology in Htra1^–/–^ mice.

**Figure 2:**
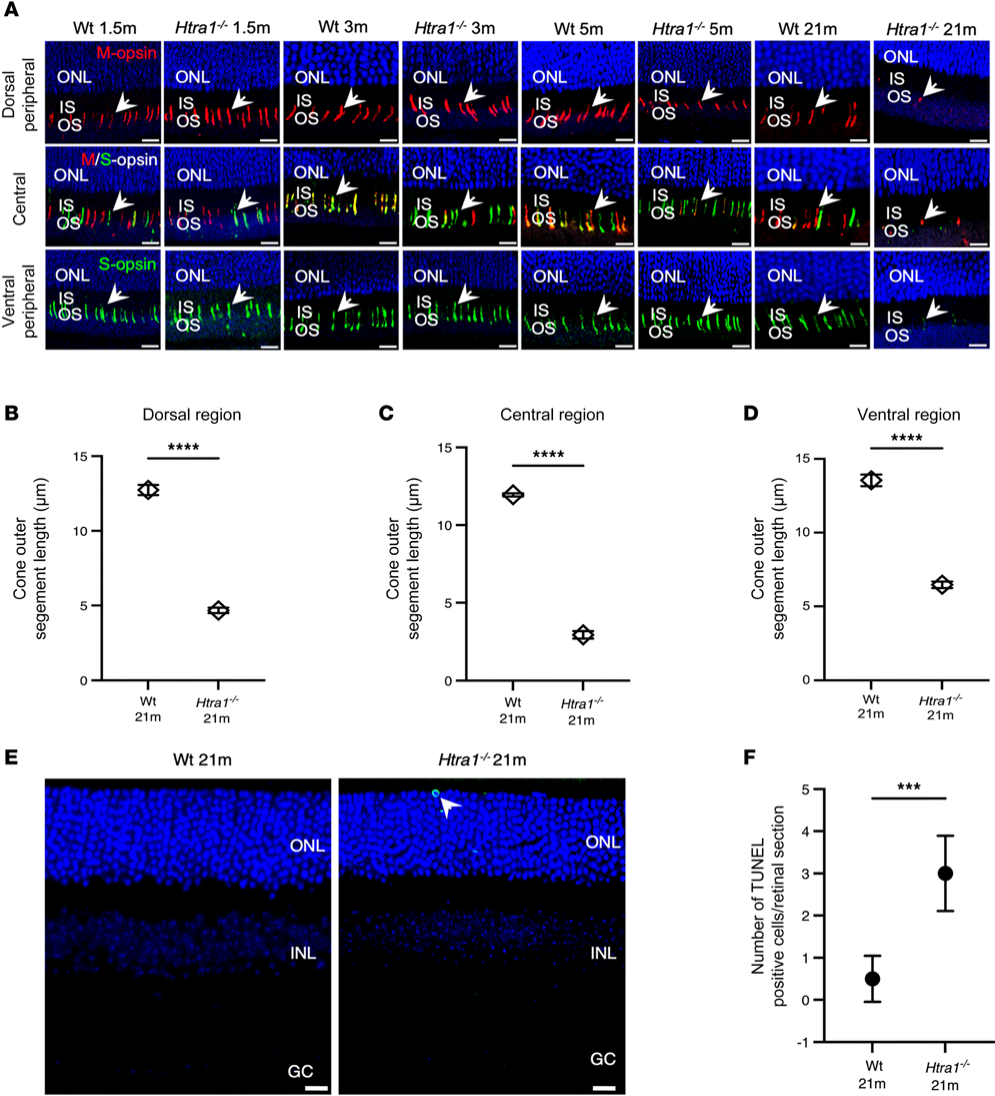
Assessment of cones and cell death in Htra1^–/–^ mice. (**A**) Representative images of M-opsin–expressing (red) and S-opsin–expressing (green) cones (white arrows) in 1.5m, 3m, 5m, and 21m WT (*n* = 4 mice per age) and *Htra1^–/–^* mice (*n* = 4 mice per age). Scale bars: 10 μm. (**B**–**D**) Cone outer segment lengths in 21m WT versus *Htra1^–/–^* mice in the dorsal, central, and ventral regions of the retina. Cone outer segment measurements were analyzed on *n* = 3 sections per genotype and averaged. Two-tailed *t* tests were performed. ****P < 0.0001. (**E** and **F**) Representative images of TUNEL assay of 21m WT and *Htra1^–/–^* mice and quantification of TUNEL^+^ cells (*n* = 3 mice per genotype). A 2-tailed *t* test was performed. ***P < 0.001. TUNEL-labeled cells are indicated by white arrow. Scale bars: 10 μm.

Because impaired cone function was observed in *Htra1^–/–^* mice from 1.5m, subsequent analysis to assess cone morphology with age was performed. Immunostaining of retinal cryosections of 1.5m, 3m, 5m, and 21m WT and *Htra1^–/–^* mice with medium wavelength (M-wavelength) and short (S-wavelength) cone opsin revealed cone abnormalities and gradual loss (Figure 2A). In *Htra1^–/–^* mice, M- and S-opsin–expressing cones in the dorsal and ventral regions, respectively, were morphologically similar to WT mice and revealed no obvious early cone degeneration at 1.5m and 3m despite perturbed cone function from 1.5m in *Htra1^–/–^* mice (Figure 2A). However, 5m *Htra1^–/–^* mice showed shorter cones in the dorsal region than WT, whereas cones in the central and ventral regions appeared normal without gross abnormalities in both models (Figure 2A). By 21m, severe cone loss was observed in the dorsal, central, and ventral regions in *Htra1^–/–^* mice (Figure 2, B–D). These results suggest a progressive cone loss with age in *Htra1^–/–^* mice. To further evaluate photoreceptor viability with age, TUNEL assay was performed on 21m WT and *Htra1^–/–^* mice. Compared with 21m WT mice, TUNEL^+^ cells were detected in the ONL of 21m *Htra1^–/–^* mice (Figure 2E). Quantification of TUNEL^+^ cells across retinal sections revealed a significant increase in 21m *Htra1^–/–^* mice (*P* = 0.0002) (Figure 2, E and F), suggesting compromised photoreceptor viability in older age *Htra1^–/–^* mice.

### Reduced photoreceptor and RPE-specific genes in Htra1^–/–^ mice.

**Figure 3:**
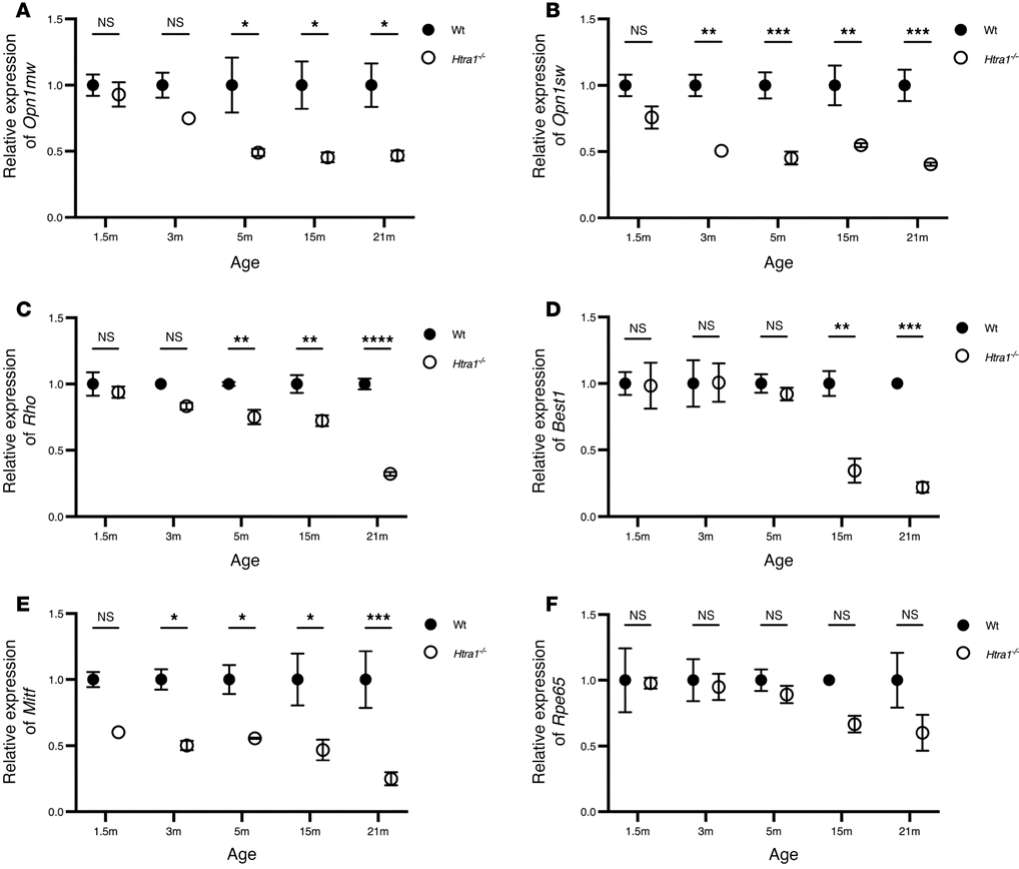
Expression of photoreceptor and RPE-specific genes in *Htra1^–/–^* mice. (**A**–**F**) *Opn1mw*, *Opn1sw*, *Rho*, *Best1*, *Mitf*, and *Rpe65* transcripts in 1.5m, 3m, 5m, 15m, and 21m *Htra1^–/–^* mice compared with age-matched WT mice (*n* = 3 mice per genotype and age point). *Actb* was used as the housekeeping control. Two-way ANOVA followed by the Tukey-Kramer for pairwise comparisons, with *P* values adjusted using the Bonferroni Correction. Age-matched comparisons are displayed. *P < 0.05, **P < 0.01, ***P < 0.001, ****P < 0.0001.

To further evaluate the physiological effect of the loss of *Htra1* on the retina, the expression of photoreceptor and RPE-specific genes were analyzed in 1.5m, 3m, 5m, 15m, and 21m *Htra1^–/–^* mice compared with either 1.5m (young age) or 21m (old age) WT controls (Figure 3, A–E). *Opn1mw* expression in 1.5m (*P* = 0.7906) and 3m (*P* = 0.0618) *Htra1^–/–^* mice was slightly decreased (Figure 3A). However, a significant reduction of *Opn1mw* was apparent at 5m (*P* = 0.0014) that progressed up to 21m (*P* < 0.0001) (Figure 3A). *Opn1sw* displayed reduced expression at 1.5m (*P* = 0.0480) that steadily progressed with age in *Htra1^–/–^* mice (Figure 3, A and B). In contrast, the expression of *Rho* was normal up to 5m (*P* = 0.0375) with decreased expression at 15m (*P* = 0.0022) and 21m (*P* < 0.0001) in *Htra1^–/–^* mice compared with 21m WT (Figure 3C). In the RPE, *Best1* expression levels were significantly reduced in 15m (*P* = 0.0015) and 21m (*P* = 0.0002) *Htra1^–/–^* mice (Figure 3D), whereas a reduction in *Mitf* expression was detected at 3m (*P* = 0.0192) and progressed up to 21m (*P* = 0.0004) (Figure 3E). Surprisingly, no significant changes were detected in *Rpe65* expression levels in *Htra1^–/–^* mice (Figure 3F).

### Htra1^–/–^ mice develop sub-RPE deposits.

**Figure 4:**
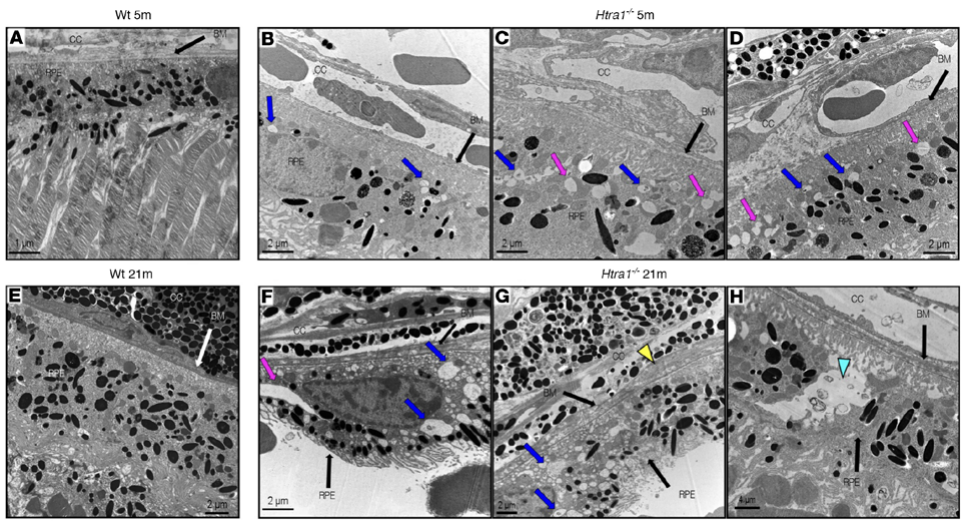
RPE abnormalities in *Htra1^–/–^* mice with age. (**A**) WT mice (5m) with choriocapillaris (CC), BM, and RPE labeled. (**B**–**D**) *Htra1^–/–^* mice (5m). Pink arrows indicate areas of vacuole-like structures. Blue arrows indicate phagosomes bearing undigested material from the photoreceptor outer segments. (**E**) WT mice (21m) with labeled CC, BM, and RPE. (**F**–**H**) *Htra1^–/–^* mice (21m). Yellow arrowhead indicates thickening of the BM. Cyan arrowhead shows a large phagosome. *n* = 3 mice per age and genotype.

**Figure 5:**
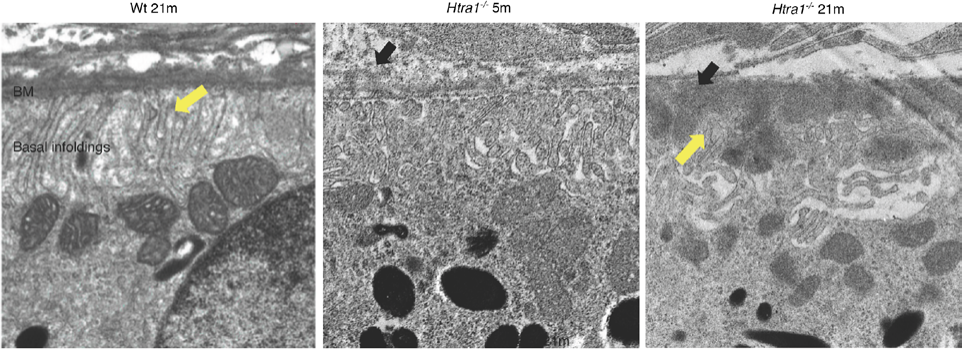
Sub-RPE deposition in *Htra1^–/–^* mice. (**A**) WT mice (21m) show intact basal infoldings (indicated by yellow arrow) and lack of RPE-BM abnormalities. (**B**) At 5m, sub-RPE deposits were present in the RPE-BM of *Htra1^–/–^* mice. (**C**) *Htra1^–/–^* mice (21m) show more sub-RPE deposits and abnormal basal infoldings. Representative images are displayed. *n* = 3 mice per age and genotype. Black arrows indicate sub-RPE deposit.

Because HTRA1 plays a major role in ECM maintenance, insight into its loss in the RPE was investigated via transmission electron microscopy. Ultrastructural analysis of 5m *Htra1^–/–^* mice revealed RPE abnormalities, including excess autophagosomes and vacuoles compared with WT mice (Figure 4, A–D). BM thickening was also apparent in 5m *Htra1^–/–^* mice (Figure 4, B–D). By 21m, RPE changes in *Htra1^–/–^* mice became more prominent as numerous small vacuoles and vesicles were detected throughout, whereas the RPE of WT mice appeared normal (Figure 4, E–H). Concomitantly, severe RPE degeneration and increased BM thickening was evident in 21m *Htra1^–/–^* mice (Figure 4, F–H). Further evaluation of the BM and sub-RPE region of the WT and *Htra1^–/–^* mice uncovered the presence of prominent BM abnormalities and sub-RPE deposits in 5m and 21m *Htra1^–/–^* mice, whereas WT mice showed minimal BM abnormalities or sub-RPE deposits (Figure 5, A–C). At 21m, basal infoldings were disrupted and filled with electron dense material and deposits in *Htra1^–/–^* mice. These results suggest that HTRA1 is essential for RPE integrity and loss of *Htra1* contributes to RPE degeneration.

### Age-related pathways in Htra1^–/–^ mice.

**Figure 6:**
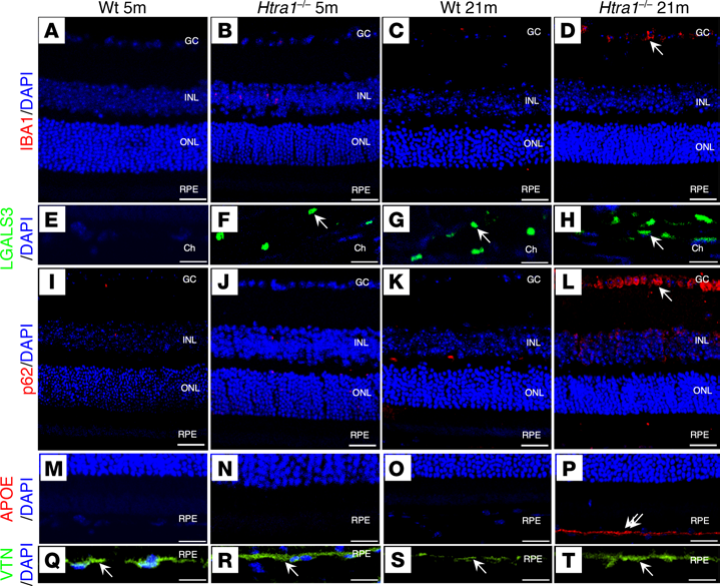
Abnormal age-related changed in *Htra1^–/–^* mice. (**A**–**T**) Representative retinal sections from 5m and 21m WT and *Htra1^–/–^* mice were stained with IBA1 (**A**–**D**), LGALS3 (**E**–**H**), p62 (**I**–**L**), APOE (**M**–**P**), and VTN (**Q**–**T**) antibodies. *n* = 3 mice per age and genotype were evaluated. DAPI was used as a counterstain. White arrows indicate signal detected. Scale bars: 50 μm.

Because of the retinal abnormalities detected in *Htra1^–/–^* mice, additional markers associated with dysregulated pathways observed in macular degenerations such as AMD were used to assess the repercussions of *Htra1* loss on the retina, RPE, and choroid. Select markers for inflammation (IBA1, Galectin-3 [LGALS3]), autophagy (p62), lipid metabolism (APOE), and ECM maintenance (vitronectin [VTN]) were evaluated in 5m and 21m WT and *Htra1^–/–^* mice (Figure 6, A–T). At 5m, IBA1 was not detected in either WT or *Htra1^–/–^* mice (Figure 6, A and B). However, expression of IBA1 was detected at 21m in *Htra1^–/–^* mice in the ganglion cell (GC) layer, but not in age-matched WT mice (Figure 6, C and D). In the choroid, LGALS3 expression was apparent in 5m *Htra1^–/–^* mice but was not detectable in 5m WT controls (Figure 6, E and F). By 21m, LGALS3 expression in *Htra1^–/–^* mice was noticeably higher than that of 21m WT mice (Figure 6, G and H). The expression of p62 was not detected in either 5m WT or *Htra1^–/–^* mice (Figure 6, I and J), but by 21m, *Htra1^–/–^* mice displayed higher levels of expression, predominantly in the GC layer (Figure 6, K and L). Similarly, APOE was not detected in either 5m *Htra1^–/–^* or WT mice (Figure 6, M and N), but accumulation of APOE in the RPE-BM of *Htra1^–/–^* mice was evident at 21m, whereas increased APOE was not detected in 21m WT mice (Figure 6, O and P). Finally, VTN showed higher levels of expression in RPE-BM of 21m *Htra1^–/–^* mice compared with WT (Figure 6, S and T), while VTN expression appeared similar between 5m WT and *Htra1^–/–^* mice (Figure 6, Q and R). These results suggest that loss of HTRA1 leads to age-related changes in key pathways associated with macular degeneration.

## Discussion

The present study characterizing the retinal phenotype in *Htra1^–/–^* mice revealed a spectrum of pathological features that progress with age in the retina and RPE. These features encompassed photoreceptor depletion and perturbed photoreceptor function, atrophy of the RPE, accumulation of sub-RPE deposits, and aberrant expression of protein markers closely linked to age-related pathogenic processes. The emergence of these distinctive pathological features as a consequence of *Htra1* deficiency underscores its pivotal function in maintaining the overall physiological integrity of both the retina and the RPE.

Photoreceptor loss is a prominent feature across retinal degenerative disorders, including AMD (30–35). Remarkably, *Htra1^–/–^* mice exhibited a substantial and progressive decline in the expression of both rod and cone photoreceptor gene markers. This decline was accompanied by a decline of photoreceptor function, and surprisingly, loss of *Htra1* perturbs cone function during early stages of pathology. The expression of *HTRA1* has been documented in photoreceptor cells alongside its established presence in the RPE (36). Interestingly, photoreceptor cell loss and progressive reduction in the expression of photoreceptor gene markers in *Htra1^–/–^* mice is similar to that of the transgenic zebrafish model that overexpresses *HTRA1* (37), suggesting that altered HTRA1 levels in the retina could contribute to photoreceptor pathology. The precise interplay between photoreceptors and *HTRA1* remains to be investigated. One possible explanation may be that *HTRA1* plays a role in photoreceptor maintenance, potentially through proteolysis of substrates expressed within photoreceptors such as TGF-β, a well-known HTRA1 substrate that promotes neuronal cell development, survival, and maturation (38–41). In this scenario, the perturbed expression of *HTRA1* may lead to unchecked TGF-β levels within photoreceptors of *Htra1^–/–^* mice, which could lead to damaging inflammatory responses and, thus, affect photoreceptor survival (42). It is also likely that the photoreceptors are affected as a consequence of the RPE abnormalities that occur due to the loss of *HTRA1*.

The presence of sub-RPE deposits in *Htra1^–/–^* mice with age suggests that the absence of HTRA1 plays a contributory role in deposit formation. These deposits, along with the observed BM thickening in *Htra1^–/–^* mice, are consistent with the hallmarks of RPE degeneration in age-related retinal pathologies such as AMD (43), DHRD/ML (44), and L-ORD (45, 46). One of the most well-established functions of HTRA1 lies in its ability to maintain ECM integrity, which comprises a vast molecular network of interactions. Secreted from the RPE, HTRA1 cleaves numerous substrates that are involved in basement membrane assembly (e.g., Nid1/2), ECM turnover (e.g., FN1), elastogenesis (e.g., FBLN5), complement activation (e.g., VTN), and amyloid deposition (e.g., A2M), among others (23, 29). Intriguingly, several ECM-associated genes implicated in deposit-forming phenotypes akin to AMD are substrates of HTRA1 (i.e., *C1QTNF5*, *EFEMP1*, *C3*) and/or are directly associated with AMD (i.e., FBLN5) (20, 21, 23, 47–49).

The precise mechanisms underlying macular degeneration remains elusive. In addition to dysregulation of ECM homeostasis, a wide array of biological processes, including inflammation (50, 51), autophagy (52, 53), oxidative stress (54, 55), and altered lipid metabolism have been postulated as potential contributors to the pathogenesis of AMD (56–59). Aberrant profiles of several markers associated with these pathways have been documented in retinal tissues of patients with AMD and animal models exhibiting deposit-forming phenotypes (60, 61). In line with these reports, our analysis unveiled an age-dependent accumulation of select markers related to these pathways in *Htra1^–/–^* mice, emphasizing the potential involvement of these pathways in retinal pathology observed due to the absence of HTRA1. Surprisingly, increased expression of p62, a marker for autophagic flux (62), was prominent in the GC layer of 21m *Htra1^–/–^* mice, and this phenomenon has been observed in age-related retinal degeneration and glaucoma models (63–65). Further studies are needed to decipher the role of p62 in *Htra1^–/–^* retinal ganglion cells with aging. Increased levels of APOE observed in 21m *Htra1^–/–^* mice, as opposed to their 5m counterparts, suggest compromised lipid trafficking and efflux in the aging RPE (66, 67). APOE has also been implicated in aging and AMD pathogenesis where it accumulates at the sites of drusen (68). VTN, one of the major components of drusen in patients with AMD (69), showed increased expression in the 21m RPE of *Htra1^–/–^* mice and is consistent with observations in additional models with sub-RPE phenotypes (60, 70, 71). Furthermore, LGALS3, an inflammatory marker that increases in the choroid of patients with dry AMD (72), and IBA1 were also upregulated in older age *Htra1^–/–^* mice. All of these findings in the *Htra1^–/–^* model are consistent with biological processes that are known to play a role in macular degeneration, including AMD (72–76). Therefore, the intricate relationship between *HTRA1* loss and its effect on these pathway warrants further investigation in the *Htra1^–/–^* model.

The role of *HTRA1* in the context of AMD has generated substantial debate, particularly concerning its expression patterns and its contribution to the development of the disease. While genome-wide association studies have identified variants within the *ARMS2/HTRA1* genetic locus that confer an elevated risk of AMD (11, 77), it remains challenging to pinpoint which of these 2 genes, *ARMS2* or *HTRA1*, is primarily responsible for AMD pathology due to their linkage disequilibrium (12, 13). Some studies have suggested that the A69S missense polymorphism in *ARMS2* increases susceptibility to AMD (12, 78). However, retinal abnormalities were not observed when the A69S *ARMS2* variant was overexpressed in mice (79). Similarly, transgenic mice overexpressing mouse *Htra1* in this study did not develop retinal abnormalities (79). In contrast, these mice displayed notable alterations in the BM, along with the development of choroidal neovascularization and sub-RPE deposits when exposed to cigarette smoke, an environmental factor associated with increased risk of AMD (79). Several studies have focused on the effect of overexpression of *HTRA1* on the RPE-BM using various models, including transgenic mice (23, 26–28, 80), zebrafish (37), and cultured RPE cells (29). Regarding the effects of increased HTRA1 expression in mice, it is worth noting that, while several transgenic mouse models were utilized to investigate retinal phenotypes, these studies did not include ultrastructural analysis of the RPE or photoreceptor morphology and functionality (27, 79, 81).

Previous studies have focused on how the expression levels of *HTRA1* contribute to AMD pathology, particularly in light of the proposition that single nucleotide polymorphisms, including rs11200638, lead to increased *HTRA1* expression in individuals with AMD (80, 82–84). However, recent reports have presented contrasting evidence of reduced levels of *HTRA1* in the RPE of AMD donor eyes from patients carrying homozygous risk variants, such as rs1049331 (24). Our findings in the *Htra1^–/–^* mice are consistent with the notion that reduced HTRA1 expression contributes to retinal and RPE pathology. It is worth noting that the reduction in *HTRA1* transcript due to the risk alleles in patients is modest in comparison with the minimal levels of transcript observed in *Htra1^–/–^* mice. Nevertheless, it is crucial to underscore that the current study on *Htra1^–/–^* mice does not negate the findings associated with *HTRA1* overexpression in disease. In fact, it is plausible that both excessive and insufficient *HTRA1* expression can have detrimental effects on the RPE. Overexpression of *HTRA1* may induce aberrant cleavage of ECM products, whereas loss of *HTRA1* could result in the accumulation of uncleaved ECM substrates, both of which could initiate events for disrupted ECM homeostasis and sub-RPE deposit formation. Thus, it is plausible that maintaining an appropriate balance of *HTRA1* is crucial for the integrity of retinal health. Further exploration into the molecular factors governing *HTRA1* during disease are necessary to improve our understanding of the relationship between *HTRA1* and retinal pathology.

To the best of our knowledge, this is the first study reporting retinal pathological features in the *Htra1^–/–^* model. Previously, studies that utilized the *Htra1^–/–^* mice did not focus on thorough characterization of the photoreceptors and RPE. Instead, these mice were evaluated for retinal polypoidal choroidal vasculopathy lesions and retinal vascular development defects (26, 41). The *Htra1^–/–^* mice have also been used to study cerebral autosomal-recessive arteriopathy with subcortical infarcts and leukoencephalopathy (CARASIL) pathogenesis where loss of *HTRA1* causes increased vascular smooth muscle cells in the thoracic aorta (85). To date, deposit-forming phenotypes in the retinas of patients with CARASIL have not been reported thus far and horizontal nystagmus was the only reported ocular phenotype in these patients (86). A possible explanation for the lack of reports of retina and RPE abnormalities in patients with CARASIL may be due to the rare prevalence of the disease, the few reported cases reported worldwide, and disease onset ~25–30 years old with reported deaths occurring within 10 years of onset (87–89).

In summary, the genetic ablation of *Htra1* in mice led to photoreceptor and RPE degeneration that progresses with age. This study allows for exploration of the functional role of *HTRA1* in photoreceptors and whether it contributes to retinal pathology in conjunction with or secondarily to abnormalities observed in the RPE-BM. The *Htra1^–/–^* mouse model serves as a valuable tool for future studies aimed at investigating the functions of *HTRA1* and deciphering molecular pathways that lead to sub-RPE deposit formation, and it allows for potential therapies that aim to restore the proper balance of *HTRA1* expression levels to combat retinal degeneration.

## Methods

### Sex as a biological variable.

Our study examined male and female mice, and no differences were found between sexes.

### Generation of the Htra1^–/–^ mouse model.

Homozygous *Htra1^–/–^* mice were previously generated on the C57BL/6J background by Chio Oka (Nara Institute of Science and Technology) (26). Briefly, part of exon 1, including ATG initiation codon, was replaced with an IRES-lacZ reporter and neomycin-resistance cassette (IRES-lacZ-neo). Genotyping of WT (C57BL/6J background, The Jackson Laboratory) and *Htra1^–/–^* mice confirmed the loss of *Htra1*. Additional screening revealed the absence of *rd1*, *rd8*, and *Rpe65* mutations in WT and *Htra1^–/–^* mice (data not shown). Furthermore, significantly reduced expression of *Htra1* transcripts were observed in *Htra1^–/–^* mice compared with control WT mice (Supplemental Figure 1). Mice were maintained in a barrier animal facility in a 12:12 light cycle at in-cage irradiance of less than 125 lux, provided standard mouse chow, and housed in a vivarium designed to meet the environmental needs for the humane care and use of the animals housed within.

### Genotyping Htra1-KO mice.

The *Htra1^–/–^* mice were screened using 2 different sets of primers (Table 1). Set A primers were designed to amplify a 412 bp segment of exon 1 present in WT, whereas Set B primers generated a 651 bp product in *Htra1^–/–^* mice due to the disruption of exon 1.

### In vivo ophthalmic imaging.

Mice were anesthetized with a mixture of ketamine (93 mg/kg body weight) (MWI Animal Health, 510189) and xylazine (8 mg/kg body weight) (VetOne, 139-236) cocktail administered i.p. During this procedure, mice were kept on a heating pad at 37°C to maintain normal body temperature. Prior to imaging, eyes were dilated with topical proparacaine hydrochloride (0.5%) (NDC 17478-263-12, Akorn Inc.) followed by topical tropicamide (0.5%) (NDC 17478-101-12, Akorn Inc.) and phenylephrine (2.5%) (NDC 17478-201-15, Akorn Inc.) administration. Spectralis HRA + OCT with Heidelberg Eye Explorer software (Heidelberg Engineering) was used for scanning laser ophthalmoscopy, infrared and spectral domain optical coherence tomography imaging. Custom-made sterilized polymethyl methacrylate contact lenses for the mice were used throughout imaging.

### ffERG.

To evaluate the retinal function, ffERGs were recorded as previously described (45) in *Htra1^–/–^* mice at 1.5m, 3m, 5m, 15m, and 21m mice. The ERG response at all ages were compared with age-matched WT mice.

### IHC.

Cryosections of WT and *Htra1^–/–^* eye globes were used to perform IHC as described previously (45). The following primary antibodies were used for staining: anti–rabbit Opsin (1:200) (MilliporeSigma, AB5405), anti–goat OPN1SW (1:200) (Santa Cruz Biotechnology, sc-14363), anti–rabbit APOE (1:100) (Abcam, ab183597), anti–rabbit IBA1 (1:200) (Fujifilm Wako Chemicals, 019-19741), anti–rabbit p62 (1:100) (MilliporeSigma, P0067), anti–goat LGALS3 (1:100) (R&D, AF1197), and anti–mouse VTN (1:50) (Santa Cruz Biotechnology, sc-74484). Secondary antibodies include donkey anti–goat Alexa Fluor 488 (Invitrogen, A11055) and donkey anti–rabbit Alexa Fluor 555 (Invitrogen, A31572). Images were captured using a ZEISS LSM 800 confocal microscope.

### TUNEL assay.

Cell death in 18m *Htra1^–/–^* retinas was detected by the In Situ Cell Death Detection Kit, Fluorescein (Roche, 11684795910) according to the manufacturer’s protocol. Slides were mounted with ProLong Gold Antifade Mountant (Thermo Fisher Scientific, P10144). Images were captured using a Keyence BZ-X Series All-in-One Fluorescence Microscope

### Quantitative PCR.

RPE and neural retina were dissected from WT and *Htra1^–/–^* mouse eyes followed by RNA isolation using the Qiagen RNeasy kit (Qiagen, 74004) and cDNA synthesis (BioRad iScript cDNA Synthesis Kit, 1708891) as previously described (21). Transcripts were detected by performing qPCR using the primers listed in Table 2.

### Transmission electron microscopy.

Eyes from WT and *Htra1^–/–^* were enucleated and fixed in 2% paraformaldehyde/2% glutaraldehyde in 0.1M sodium cacodylate buffer, pH 7.4, overnight at 4°C. Fixation was quenched in 100 mM glycine in PBS, pH 7.4, for 10 minutes at room temperature and subsequently washed in 0.1M sodium cacodylate buffer, pH 7.4. After cornea dissections, eye cups were washed in 0.1M sodium cacodylate buffer, pH 7.4, and post-fixed in 1% osmium tetroxide for 1 hour at room temperature. After osmication, tissues were washed in 0.1M sodium cacodylate buffer, pH 7.4, followed by distilled water before dehydration with incubations in a graded ethanol series (50%, 70%, 85%, 95%, and 100%). Tissues were then incubated in a transitional solvent of 1:1 ethanol/propylene oxide followed by 100% propylene oxide and overnight infiltration in 1:1 propylene oxide/Embed 812 resin. The following day, tissues were further infiltrated in 100% Embed 812 resin for 2 hours. After infiltration, tissues were flat embedded in Embed 812 resin and baked for 48 hours at 65°C. Once embedded, tissues were sectioned at 65 nm. Sections were stained using Uranyless stain (Electron Microscopy Sciences) and lead citrate (Electron Microscopy Sciences). Sections were imaged on a JEOL 2000EX Electron Microscope and analyzed for ultrastructural abnormalities.

### Statistics.

Two-way ANOVA was used to evaluate WT vs *Htra1^–/–^* at designated age points. A post-hoc analysis was implemented to evaluate pairwise comparisons using the Tukey-Kramer test. Comparisons strictly between age-matched *Htra1^–/–^* and WT, with associated significance levels, are displayed in the figures. Significance levels have been adjusted for multiple comparisons using the Bonferroni correction. GraphPad Prism software (version 10.0.1). Data analyses between WT and *Htra1^–/–^* were performed by 2-tailed *t* test. Significance was set at *P < 0.05, **P < 0.01, ***P < 0.001 and ****P < 0.0001 in GraphPad Prism.

### Study approval.

All animal protocols were approved by the IACUC at the UCSD.

### Data availability.

All raw data for this manuscript are provided in the Supporting Data Values file.

## Author contributions

PB designed the study, performed experiments, analyzed data, and wrote the manuscript. DRW performed experiments, analyzed data, and wrote the manuscript. TJH performed experiments and analyzed data. NWK analyzed data and contributed to interpretation of the data. DRL, AMB, and MD contributed to data acquisition and analysis. YP, DG, PXS, CO, and TI contributed to manuscript preparation. MMJ contributed to data analysis and manuscript preparation. RA designed the study and contributed to data interpretation and manuscript preparation.

## Supplementary Material

Supplemental data

Unedited blot and gel images

Supporting data values

## Figures and Tables

**Table 1:**
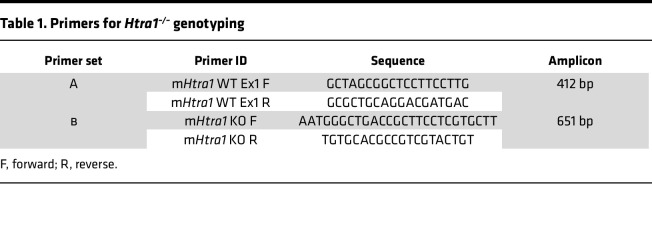
Primers for *Htra1^–/–^* genotyping

**Table 2:**
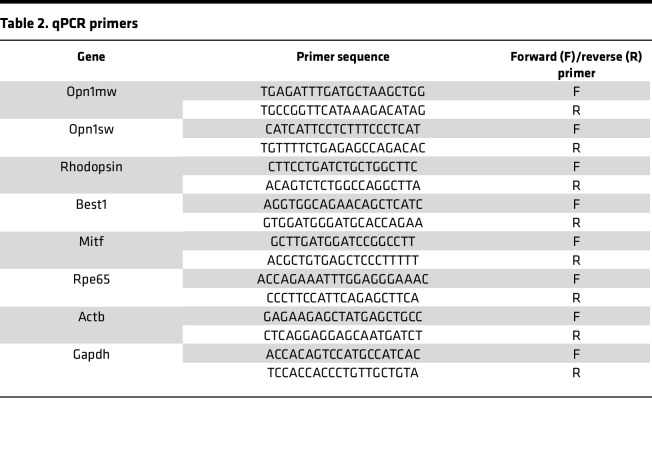
qPCR primers

## Version 1 02/10/2025

Electronic publication
