# Supplementary material for: Ablation of Htra1 leads to sub-RPE deposits and photoreceptor abnormalities
Source: JCI Insight. 2025 Feb 10;10(3):e178827. doi: 10.1172/jci.insight.178827 (PMC11948579; doi:10.1172/jci.insight.178827)
Supplement: Supplemental data [file jciinsight-10-178827-s179.pdf]

Supplementary Figure 1. *Htra1$^{-/-}$* mouse model generation and *Htra1* expression.

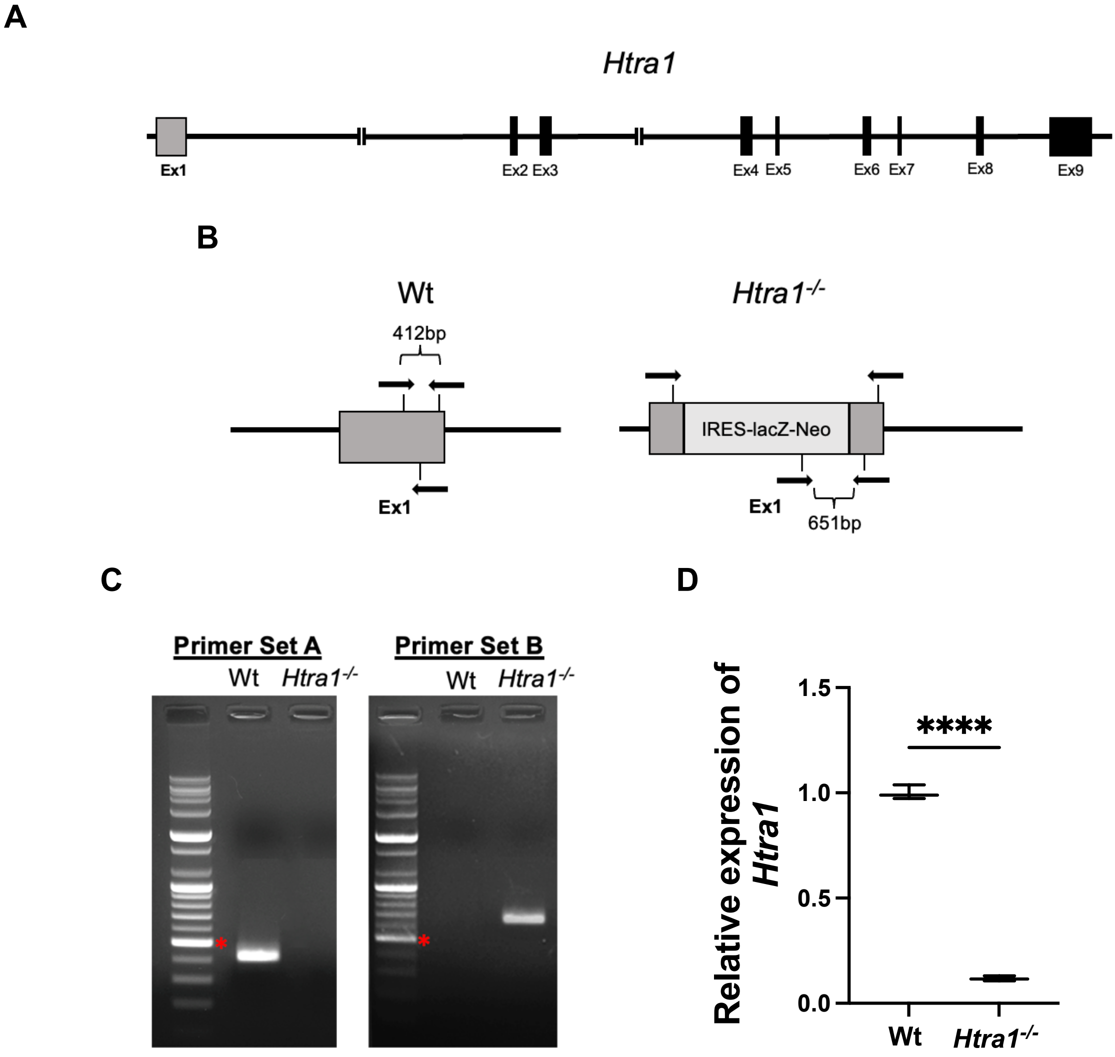

(A) Schematic diagram of the *Htra1* gene. Exon 1 is highlighted in grey. (B) Generation of *Htra1$^{-/-}$* mice and location of primer binding sites for genotyping exon 1 of the Wt and *Htra1$^{-/-}$* alleles. Black arrow indicates primer. (C) Primer Set A amplified a 412bp product for Wt whereas Primer Set B amplified a 412bp product for *Htra1$^{-/-}$* mice. Red asterisk = 500bp. (D) qPCR confirmation of significantly low levels of *Htra1* mRNA in the *Htra1$^{-/-}$* mouse model.

Supplementary Figure 2. Dark-adapted and light-adapted ERG responses in *Htra1$^{-/-}$* mice.

A

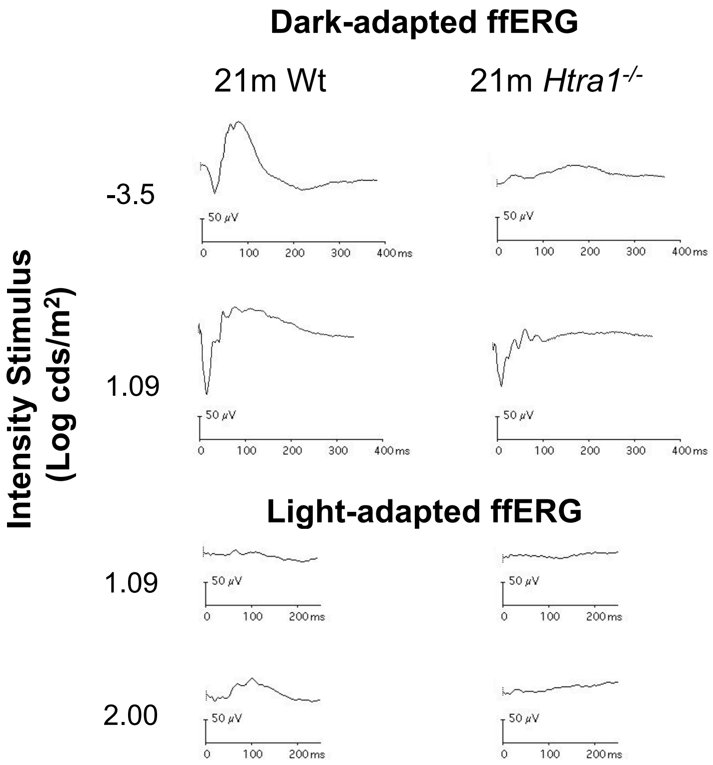

(A) Dark-adapted ERG shows the rod-mediated response and the combined rod-cone response. Light-adapted ERG shows the cone-mediated response.
