# Supplementary figures and images for: Ablation of Htra1 leads to sub-RPE deposits and photoreceptor abnormalities
Source: JCI Insight. 2025 Feb 10;10(3):e178827. doi: 10.1172/jci.insight.178827 (PMC11948579; doi:10.1172/jci.insight.178827)

**Primer set A**

Wt    *Htra1*<sup>-/-</sup>

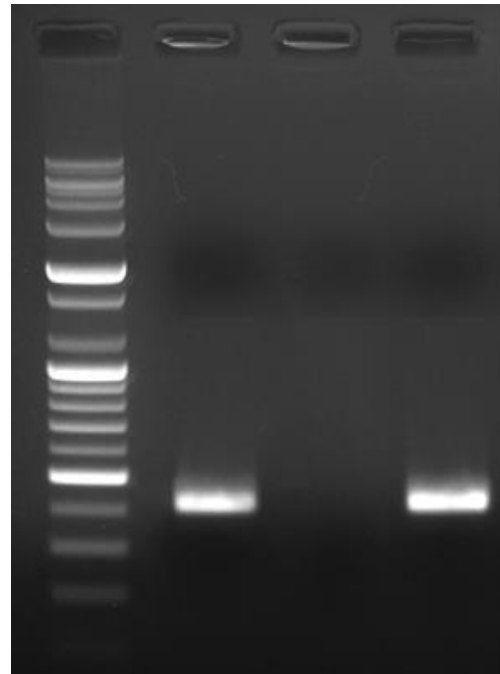

**Primer set B**

Wt    *Htra1*<sup>-/-</sup>

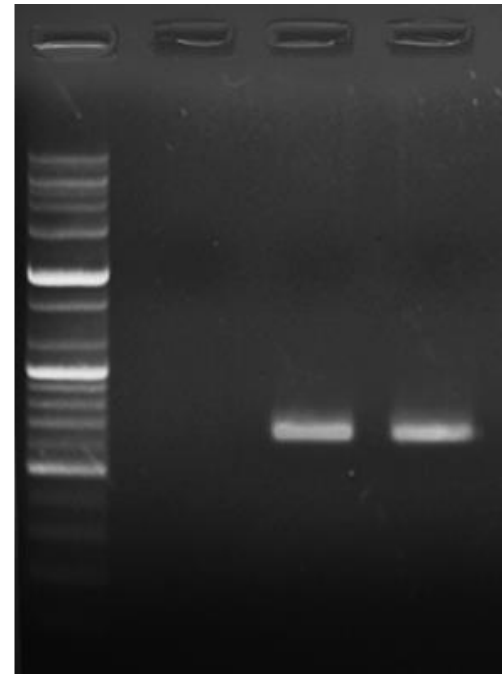

Supplement: Unedited blot and gel images [file jciinsight-10-178827-s180.pdf]
